# An immune‐related seven‐lncRNA signature for head and neck squamous cell carcinoma

**DOI:** 10.1002/cam4.3756

**Published:** 2021-03-03

**Authors:** Yue Chen, Tian‐Qi Luo, Si‐Si Xu, Chun‐Yan Chen, Ying Sun, Li Lin, Yan‐Ping Mao

**Affiliations:** ^1^ Department of Radiation Oncology Sun Yat‐sen University Cancer Center State Key Laboratory of Oncology in South China Collaborative Innovation Center of Cancer Medicine Guangdong Key Laboratory of Nasopharyngeal Carcinoma Diagnosis and Therapy Guangzhou Guangdong People's Republic of China; ^2^ Department of Gastric Surgery Sun Yat‐sen University Cancer Center State Key Laboratory of Oncology in South China Guangzhou Guangdong People's Republic of China

**Keywords:** head and neck squamous cell carcinoma, immune, lncRNA, tumor microenvironment

## Abstract

In this study, we developed a long noncoding RNA (lncRNA)‐based prognostic signature for stratification of patients with head a nd neck squamous cell carcinoma (HNSCC). In total, 493 HNSCC samples obtained from the Cancer Genome Atlas database were divided into training and testing cohorts (3:2 ratio). We identified 3913 immune‐related lncRNAs in the HNSCC training cohort by Pearson correlation analysis; only seven were independently associated with overall survival and were used to develop an immune‐related lncRNA prognostic signature (IRLPS) grouping of HNSCC patients into high‐ and low‐IRLPS subgroups. Univariate and multivariate Cox analyses revealed that low‐IRLPS patients had a better prognosis in all the cohorts, which was retained after stratification by sex, grade, and HPV status. Although the TNM stage was also an independent prognostic factor, the IRLPS had a better discriminability with higher AUC at the 3‐ and 5‐year follow‐ups in all cohorts. Low‐IRLPS samples had more immune cell infiltration and were enriched in immune‐related pathways, while high‐ IRLPS samples were enriched in metabolic pathways. A nomogram constructed including age, TNM stage, and IRLPS showed good calibration. Thus, IRLPS improves the prognostic prediction and also distinguishes different tumor microenvironment (TME) in HNSCC patients.

## Graphical abstract

We developed an immune‐related lncRNA signature for head and neck squamous cell carcinoma (HNSCC) patients, which could improve the prognostic prediction and distinguish different tumor microenvironments (TMEs) in HNSCC patients.

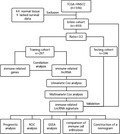

## 1 INTRODUCTION

Head and neck cancers are the third most commonly diagnosed cancers (8% of the total cases) and the seventh leading causes of cancer deaths worldwide (5.2% of the total cancer deaths) according to GLOBOCAN 2018.^1^ Head and neck squamous cell carcinoma (HNSCC) is the most common tumor type of head and neck cancers.^2^ Although the diagnosis and treatment of HNSCC continue to improve, the 5‐year survival rate is still <50%.^3^ Improving prognostic prediction and developing individualized treatments are vital measures to improve HNSCC patient prognosis. TNM stage based on anatomic factors is a crucial factor for guiding treatment options, but not all patients with the same TNM stage present similar treatment outcomes because of molecular heterogeneity, so its prediction ability is limited.^4^, ^5^ Thus, there is an urgent need to discover sensitive and specific molecular markers to predict the prognosis of HNSCC patients.

In the previous studies, researchers have identified signatures of HNSCC based on protein expression,^6^ immune‐related gene expression,^7^ genetic variants,^8^ chemokines,^9^ DNA methylation,^10^ and mRNA expression.^11^ Long noncoding RNAs (lncRNAs) are involved in the process of tumor development including tumorigenesis and metastasis.^12^, ^13^ Furthermore, lncRNAs play a vital role in the tumor microenvironment (TME) and are correlated with patient prognosis.^14^, ^15^, ^16^, ^17^ However, the effects of immune‐related lncRNAs in the TME and predicting the prognosis of HNSCC remain unknown.

In our study, we developed an immune‐related lncRNA prognostic signature (IRLPS) for HNSCC based on the Cancer Genome Atlas (TCGA) training cohort and evaluated its prognostic role in the testing and entire cohorts and different groups. We also compared the discriminability between the IRLPS score and the TNM stage and the distribution of immune cells and functional enrichment in different IRLPS subgroups. We found that IRLPS was a reliable prognostic biomarker and could be used to distinguish different TME characteristics of HNSCC.

## 2 MATERIALS AND METHODS

### 2.1 Clinical samples and data sets

Transcriptome sequencing data of 493 HNSCC samples (excluding 44 normal samples and nine HNSCC samples without survival information) and their survival information were downloaded from the TCGA database (https://portal.gdc.cancer.gov/projects/TCGA‐HNSC). The 493 HNSCC patients were randomly divided (3:2 ratio) into a training cohort (*n* = 297) to identify a prognostic lncRNA signature and build a prognostic IRLPS model, and a testing cohort (*n* = 196) for validating its prognostic value. The lists of immune‐related genes (IMMUNE_RESPONSE and IMMUNE_SYSTEM_PROCESS gene sets) were obtained from the Gene Set Enrichment Analysis (GSEA) database (https://www.gsea‐msigdb.org/gsea/downloads.jsp).

### 2.2 Immune‐related lncRNAs and survival analysis

The genes of the TCGA training cohort (*n* = 297) included in the gene sets of the IMMUNE_RESPONSE or IMMUNE_SYSTEM_PROCESS from the GSEA database were considered to be immune‐related genes. LncRNAs associated with the immune‐related genes were identified by Pearson correlation analysis with a threshold *r* > 0.4. Next, we performed univariate and multivariate Cox analyses on the immune‐related lncRNAs to identify the prognostic lncRNAs with the survival package of R. The correlations between the prognostic lncRNAs that were found to be significant in the univariate Cox analysis were analyzed with the Hmisc package of R.

### 2.3 Construction of an IRLPS

To construct an IRLPS, the score of each sample in the TCGA training cohort was calculated as the sum of the expression values of each lncRNA multiplied by their weight in the multivariate Cox model. Based on the calculated IRLPS score and IRLPS, the patients in the training cohort were distributed into high‐ and low‐IRLPS subgroups based on a median IRLPS score cutoff value. The distribution of the IRLPS score along with the expression level of seven lncRNAs and the corresponding survival status were analyzed in the two IRLPS subgroups with gene expression heat maps.

### 2.4 Prognostic value of IRLPS

To estimate the prognostic power of IRLPS for overall survival (OS) time, Kaplan–Meier (K–M) survival curves with log‐rank tests were performed on the two IRLPS subgroups in the training cohort and validated in the testing and entire cohorts.

Besides, we constructed time‐dependent receiver operating characteristic (ROC) curves with the 3‐ and 5‐year follow‐ups as the defining point. We then calculated the area under the ROC curve (AUC) to evaluate the predictive power of IRLPS in the training cohort with the timeROC package of R. The results were validated in the testing and entire cohorts.

### 2.5 Univariate and multivariate analyses of different clinicopathological factors

To determine the independent prognostic clinicopathological factors for HNSCC, we conducted a univariate Cox analysis of the IRLPS score, age, sex, smoking, alcohol history, HPV status, grade, and TNM stage. Factors found to be significant in the univariate Cox analysis were included in multivariate Cox models. To study the relationship between the IRLPS score and other clinicopathological factors, Wilcoxon tests were used to compare the IRLPS scores between the two subgroups of different clinicopathological factors.

### 2.6 ROC analysis of IRLPS score and TNM stage

To compare the discriminability between the TNM stage and IRLPS score of IRLPS, we constructed time‐dependent ROC curves of stage and IRLPS score at the 3‐ and 5‐year follow‐ups in the training cohort with the timeROC package of R. The results were validated in the testing and entire cohorts.

### 2.7 Survival analysis in the different subgroups

To further evaluate the role of the IRLPS score in distinguishing the prognosis of HNSCC patients, we constructed K–M survival curves and conducted log‐rank tests in the groups stratified according to all the related clinicopathological factors, including sex, grade, and HPV status.

### 2.8 Mutation status in two IRLPS subgroups

To study the mutation status of the HNSCC sample, the top 10 genes with the highest mutation rate in the two different IRLPS subgroups were shown using the maftools package of R.

### 2.9 Comparison of immune cell infiltration in two IRLPS subgroups

To study the role of IRLPS in distinguishing the TME of HNSCC, the distribution of six immune cell types was compared between the two different IRLPS subgroups by the Wilcoxon test using the Timer (https://cistrome.shinyapps.io/timer/) database.

### 2.10 Functional enrichment analysis

We performed GSEA of the high‐ and low‐IRLPS subgroups separated by IRLPS score with the gene sets of the Gene Ontology (GO) terms and Kyoto Encyclopedia of Genes and Genomes (KEGG) pathways. Both the false discovery rate (FDR) value <0.25 and the nominal *p*‐value <0.05 were used to sort the pathways enriched in each phenotype.^18^, ^19^ Single sample GSEA (ssGSEA) analysis was then performed on several representative gene sets with the GSVA package of R, and K–M survival curves were used to explore differences in survival.

### 2.11 Nomogram construction

To individualize the predicted 3‐ and 5‐year survival probability, a nomogram was constructed based on the results of the multivariate analysis. The nomogram included significant clinicopathological characteristics and tested its predictive accuracy with the calibration plot with the rms package of R.

### 2.12 Statistical analysis

All statistical tests were two‐tailed with a statistical significance level set at 0.05. Wilcoxon tests were performed for comparison of continuous variables between the two IRLPS subgroups. Categorical data were tested with the chi‐squared test.

## 3 RESULTS

### 3.1 Immune‐related lncRNAs in HNSCC

**FIGURE 1:**
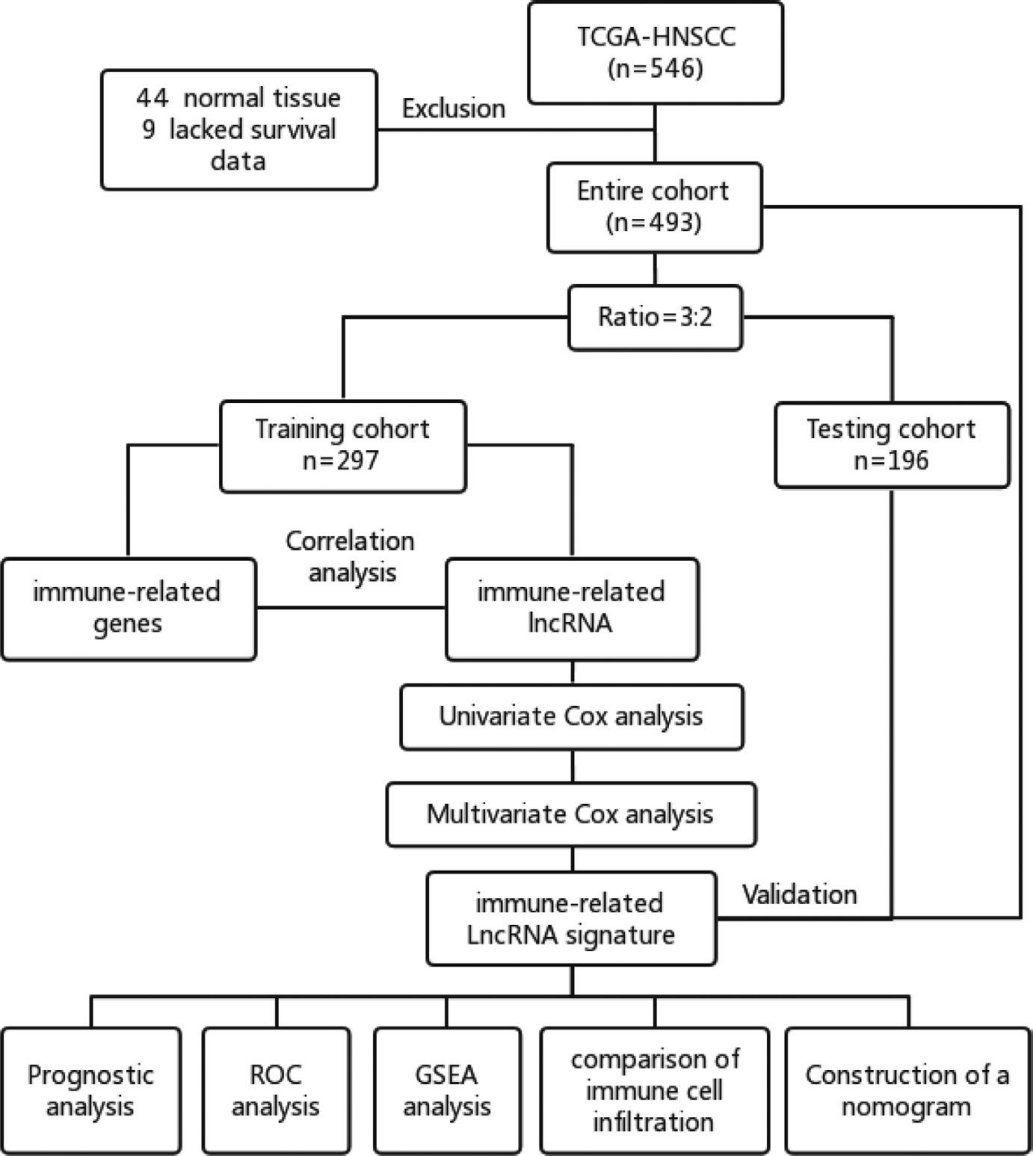
Flowchart of data analysis

A total of 493 HNSCC samples were obtained from the TCGA database. These samples were randomly distributed into training and testing cohorts at a ratio of 3:2 as shown in the flowchart in Figure 1. The 297 HNSCC samples in the training cohort were used to construct an immune‐related lncRNA signature and a prognostic model, while 196 HNSCC samples in the testing cohort were used to evaluate the performance of IRLPS and the prognostic model. Details of the HNSCC samples in the training and testing cohorts are displayed in Table 1. Based on the gene lists of the training cohort, 260 immune‐related genes were included in the gene lists of IMMUNE_RESPONSE or IMMUNE_SYSTEM_PROCESS gene sets from the GSEA database. A total of 3913 immune‐related lncRNAs were identified in the training cohort by Pearson correlation analysis with thresholds *r* > 0.4 and *p* < 0.05.

**TABLE 1:** The clinicopathological factors of head and neck squamous cell carcinoma (HNSCC) samples

Risk groups	The whole cohort			Training cohort			Testing cohort		
	Training cohort *n* = 287	Testing cohort *n* = 196	*p*	High risk *n* = 148	Low risk *n* = 149	*p*	High risk *n* = 89	Low risk *n* = 107	*p*
Risk score (95% CI)	0.98 [0.67, 1.56]	0.93 [0.59, 1.43]	0.118	1.56 [1.22, 2.17]	0.67 [0.47, 0.80]	<0.001	1.48 [1.20, 1.86]	0.62 [0.45, 0.83]	<0.001
Age (median [IQR])	61 [53, 69]	60 [54, 67]	0.486	64 [53, 73]	60 [53, 67]	0.019	61 [52, 67]	60 [54, 69]	0.686
Sex (%)			0.904			1			0.232
Male	217 (73.1)	145 (74.0)		108 (73.0)	109 (73.2)		70 (78.7)	75 (70.1)	
Female	80 (26.9)	51 (26.0)		40 (27.0)	40 (26.8)		19 (21.3)	32 (29.9)	
Smoking			0.030			0.065			0.122
No	75 (25.3)	33 (16.8)		30 (20.3)	45 (30.2)		11 (12.4)	22 (20.6)	
Yes	214 (72.0)	161 (82.1)		112 (75.7)	102 (68.5)		78 (87.6)	83 (77.6)	
Unknown	8 (2.7)	2 (1.1)		6 (4.0)	2 (1.3)		0 (0.0)	2 (1.8)	
Alcohol history			0.525			0.449			0.365
No	97 (32.7)	55 (28.1)		53 (35.8)	44 (29.5)		27 (30.3)	28 (26.2)	
Yes	193 (65.0)	137 (69.9)		91 (61.5)	102 (68.5)		59 (66.3)	78 (72.9)	
Unknown	7 (2.3)	4 (2.0)		4 (2.7)	3 (2.0)		3 (3.4)	1 (0.9)	
HPV status (%)			0.081			0.014			0.211
Negative	50 (16.8)	21 (10.7)		27 (18.2)	23 (15.4)		9 (10.1)	12 (11.2)	
Positive	15 (5.1)	16 (8.2)		2 (1.4)	13 (8.7)		4 (4.5)	12 (11.2)	
Unknown	232 (78.1)	159 (81.1)		119 (80.4)	113 (75.8)		76 (85.4)	83 (77.6)	
Grade (%)			0.136			0.056			0.048
I	40 (13.5)	21 (10.7)		21 (14.2)	19 (12.8)		5 (5.6)	16 (15.0)	
II	168 (56.5)	126 (64.3)		93 (62.8)	75 (50.3)		66 (74.2)	60 (56.1)	
III	78 (26.3)	39 (19.9)		31 (20.9)	47 (31.5)		14 (15.7)	25 (23.3)	
IV	0 (0.0)	2 (1.0)		0 (0)	0 (0)		0 (0.0)	2 (1.9)	
Unknown	11 (3.7)	8 (4.1)		3 (2.1)	8 (5.4)		4 (4.5)	4 (3.7)	
TNM stage (%)			0.301			0.596			0.038
I	18 (6.1)	7 (3.6)		6 (4.1)	12 (8.0)		5 (5.6)	2 (1.9)	
II	44 (14.8)	25 (12.8)		22 (14.9)	22 (14.8)		11 (12.4)	14 (13.1)	
III	46 (15.5)	32 (16.3)		24 (16.2)	22 (14.8)		13 (14.6)	19 (17.8)	
IV	143 (48.1)	110 (56.1)		75 (50.7)	68 (45.6)		56 (62.9)	54 (50.4)	
Unknown	46 (15.5)	22 (11.2)		21 (4.1)	25 (16.8)		4 (4.5)	18 (16.8)	
Survival status (%)			0.826			<0.001			0.092
Dead	130 (43.8)	83 (42.3)		85 (57.4)	45 (30.2)		44 (49.4)	39 (36.4)	
Alive	167 (56.2)	113 (57.7)		63 (42.6)	104 (69.8)		45 (50.6)	68 (63.6)	
Survival time (95% CI)	1.73 [1.03, 3.14]	1.78 [1.08, 3.25]	0.687	1.48 [0.76, 2.53]	2.18 [1.16, 4.17]	<0.001	1.64 [1.10, 2.96]	1.92 [1.07, 3.77]	0.175

### 3.2 Development of an immune‐related lncRNA signature

**FIGURE 2:**
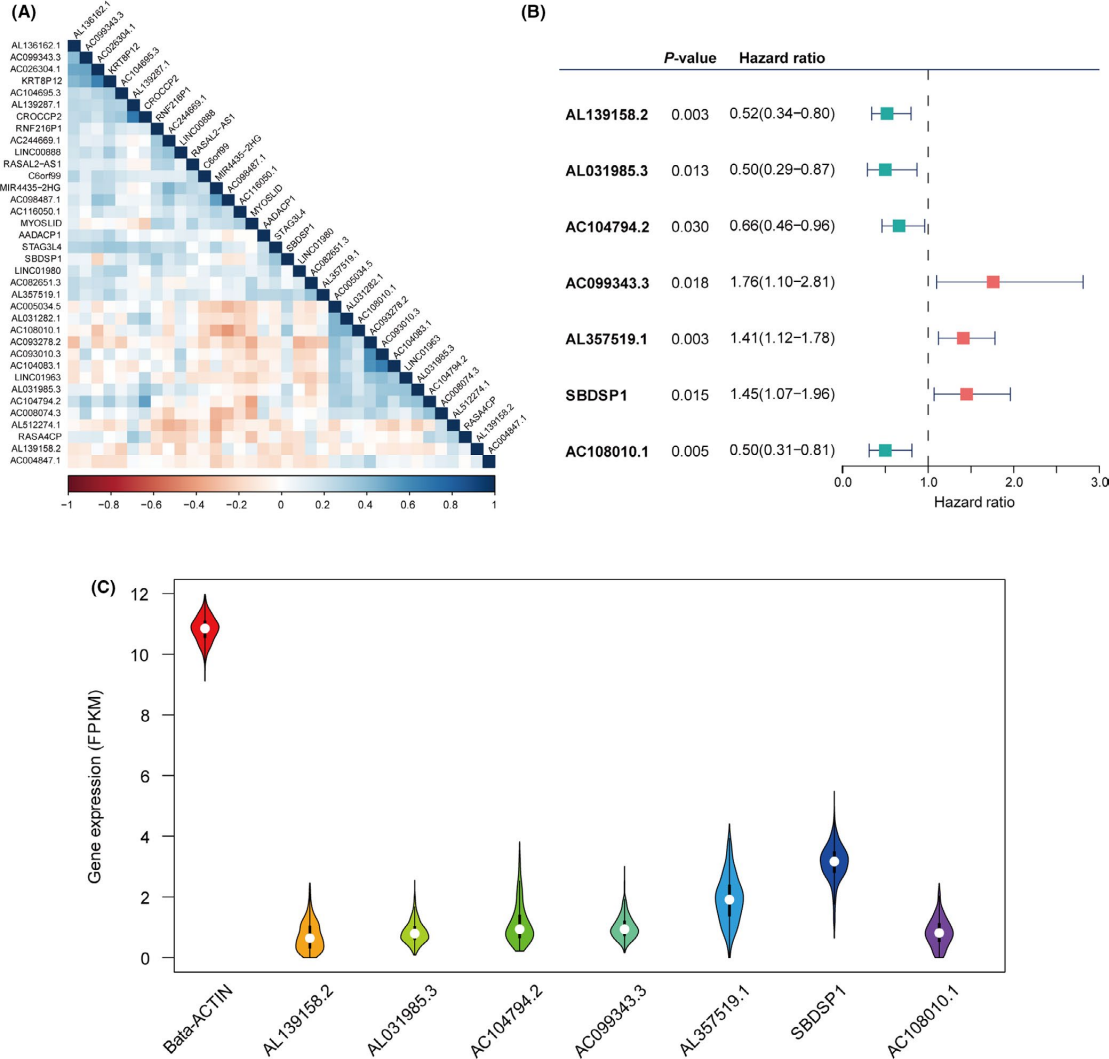
Identification of the immune‐related long noncoding RNA (lncRNA) signature. (A) Heat map of the correlations of the prognostic immune‐related lncRNAs. (B) Multivariate Cox model based on the immune‐related lncRNA signature. (C) The relative expression of seven lncRNAs with beta‐actin as the internal reference

To find out the prognostic lncRNAs, we carried out univariate and multivariate Cox analysis on 3,913 immune‐related lncRNA. As a result, 36 lncRNA affected patient OS in the univariate Cox analysis (*p* all <0.05); the correlations are shown in Figure 2A. After the inclusion of these candidate prognostic lncRNAs put in the multivariable Cox model, we found only seven lncRNAs that were independent survival‐related lncRNAs (*p* all <0.05) (Figure 2B). The details of the seven lncRNAs are shown in Table S1. In addition, the relative expression level of the seven lncRNAs with beta‐actin used as the internal reference is shown in Figure 2C. Furthermore, an immune‐related lncRNA prognostic biomarker was developed based on these seven lncRNAs, and the IRLPS score was calculated as the formula IRLPS score = expression of AL139158.2*(−0.652) + expression of AL031985.3*(−0.687) + expression of AC104794.2*(−0.414) + expression of AC099343.3*(0.565) + expression of AL357519.1*(0.346) + expression of SBDSP1*(0.372) + expression of AC108010.1*(−0.691).

**FIGURE 3:**
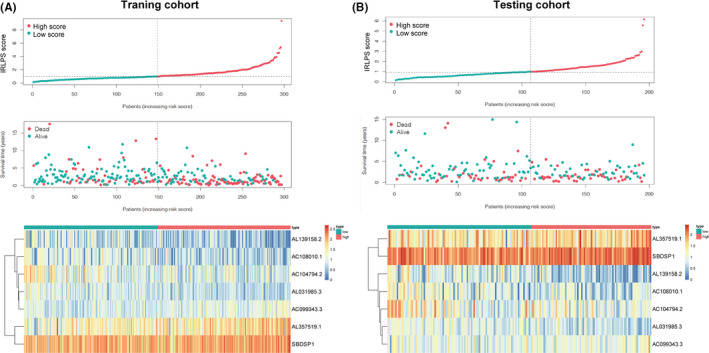
The characteristics of the high‐ and low‐immune‐related lncRNA prognostic signature (IRLPS) subgroups. Distribution of the IRLPS score along with the corresponding survival status and the expression level of seven long noncoding RNAs (lncRNAs) in the two IRLPS subgroups in the (A) training cohort and (B) testing cohort

HNSCC patients were divided into low‐ and high‐IRLPS subgroups based on the median value of the IRLPS score. The distribution of the IRLPS score along with the expression level of seven lncRNA and the corresponding survival status for the two IRLPS subgroups in the training and testing cohorts are displayed in Figure 3A,B, respectively.

### 3.3 Prognostic role of IRLPS

**FIGURE 4:**
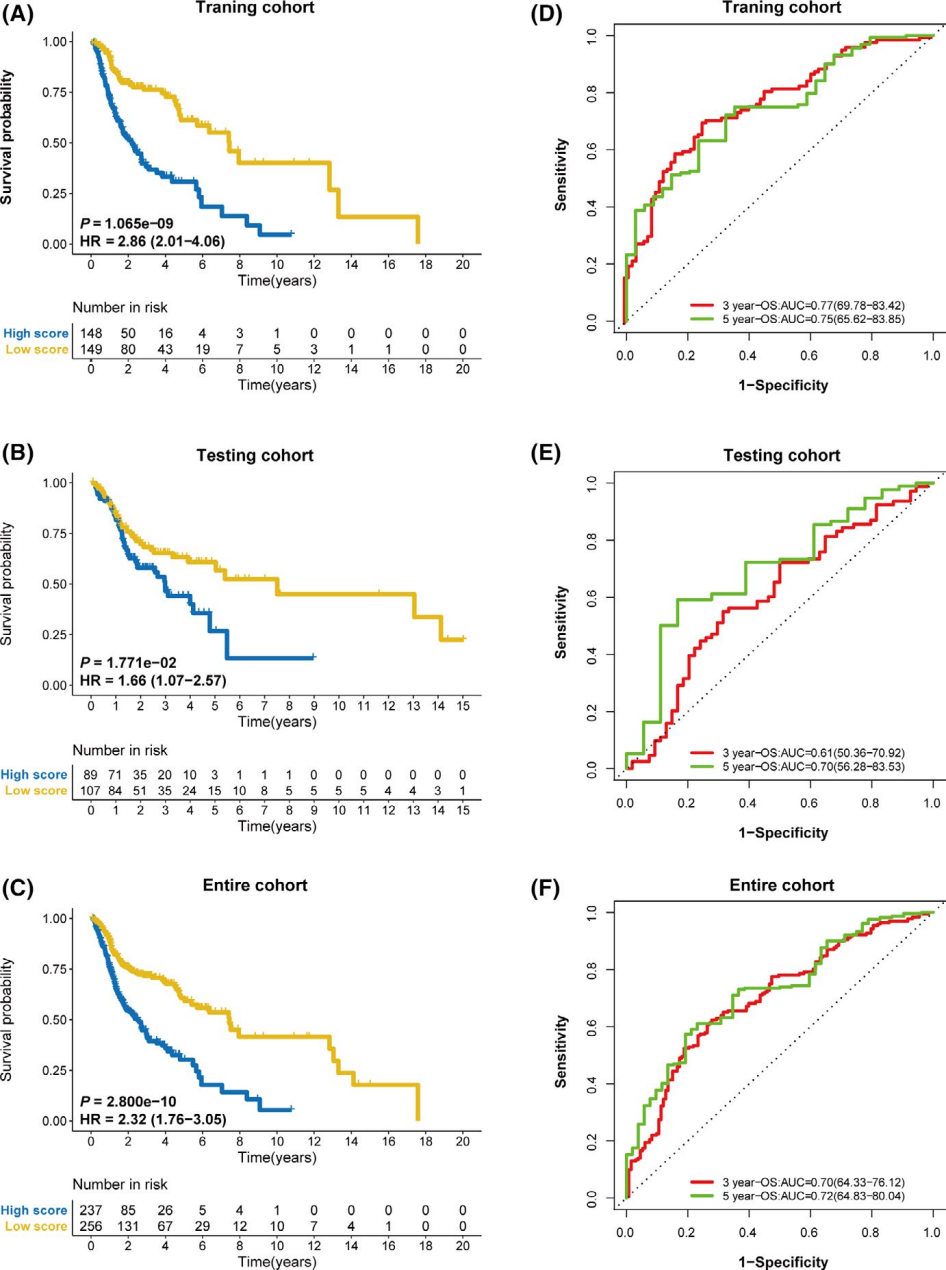
The prognostic role of immune‐related lncRNA prognostic signature (IRLPS). Kaplan–Meier survival curves for overall survival of the two IRLPS subgroups in the (A) training cohort, (B) testing cohort, and (C) entire cohort. The ROC curves for overall survival at the 3‐ and 5‐year follow‐ups in the (D) training cohort, (E) testing cohort, and (F) entire cohort

To clarify the role of IRLPS in predicting patient outcomes, K–M survival curves were constructed and log‐rank tests were performed. As shown in Figure 4A, we found that the low‐IRLPS group had a longer OS than the high‐IRLPS group in the training cohort (*p* = 1.065 × 10^−9^). This result was consistent with that of the testing cohort (*p* = 1.771 × 10^−2^) and the entire cohort (*p* = 2.800 × 10^−10^), shown in Figure 4B,C. Also, we performed a time‐dependent ROC analysis to evaluate the prognostic value of IRLPS. The AUC value of the ROC curve analysis of the prognostic signature was 0.77 and 0.75 at the 3‐ and 5‐year follow‐ups, respectively, in the training cohort (Figure 4D), 0.61 and 0.70 in the testing cohort (Figure 4E), and 0.70 and 0.72 in the entire cohort, respectively (Figure 4F).

### 3.4 Independent prognostic factors for OS

As shown in Table 1, the clinical and prognostic factors including risk scores, age, sex, smoking, alcohol history, HPV status, grade, TNM stage, survival status, and survival time were all evenly distributed between the training cohort and the testing cohort, except smoking. Due to the uneven distribution of smoking in the two cohorts, it was necessary to explore whether smoking was an important factor affecting the prognosis of HNSCC patients. As a result, smoking was not a significant prognostic factor of both the training cohort and the testing cohort in the univariate Cox analysis.

**FIGURE 5:**
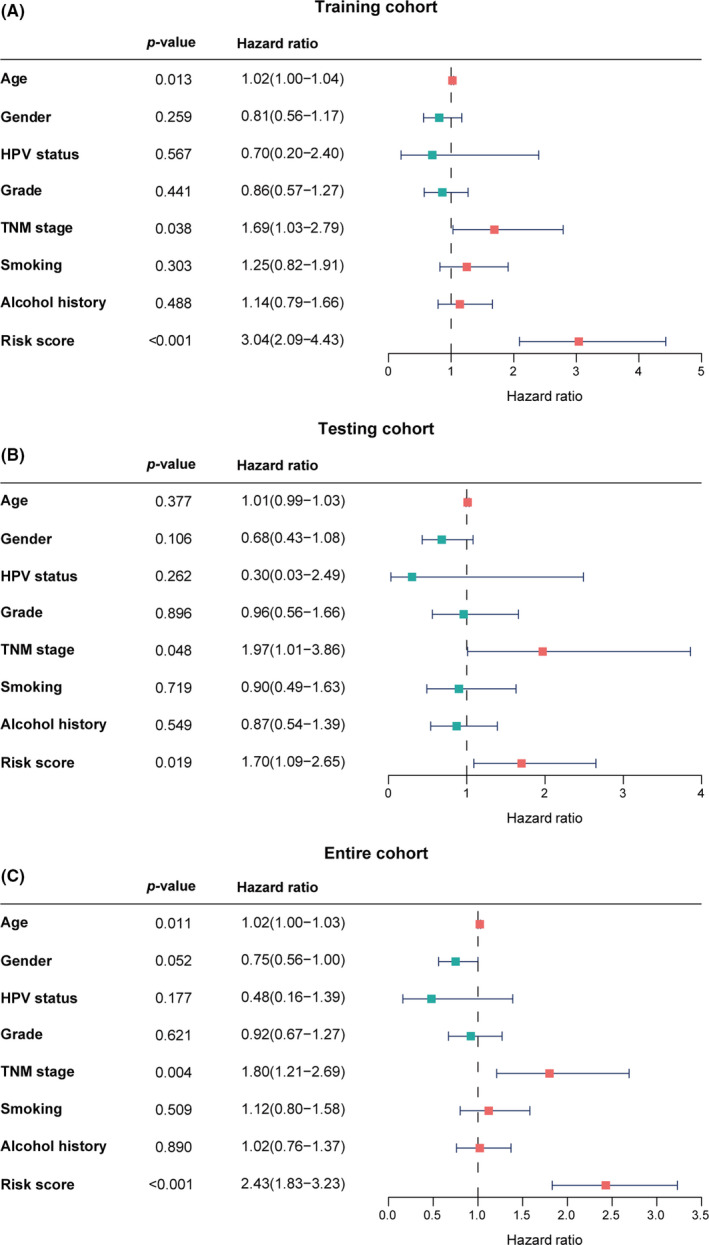
Significant prognostic factors for overall survival. Univariate analysis of the (A) training cohort, (B) testing cohort and, (C) entire cohort

**FIGURE 6:**
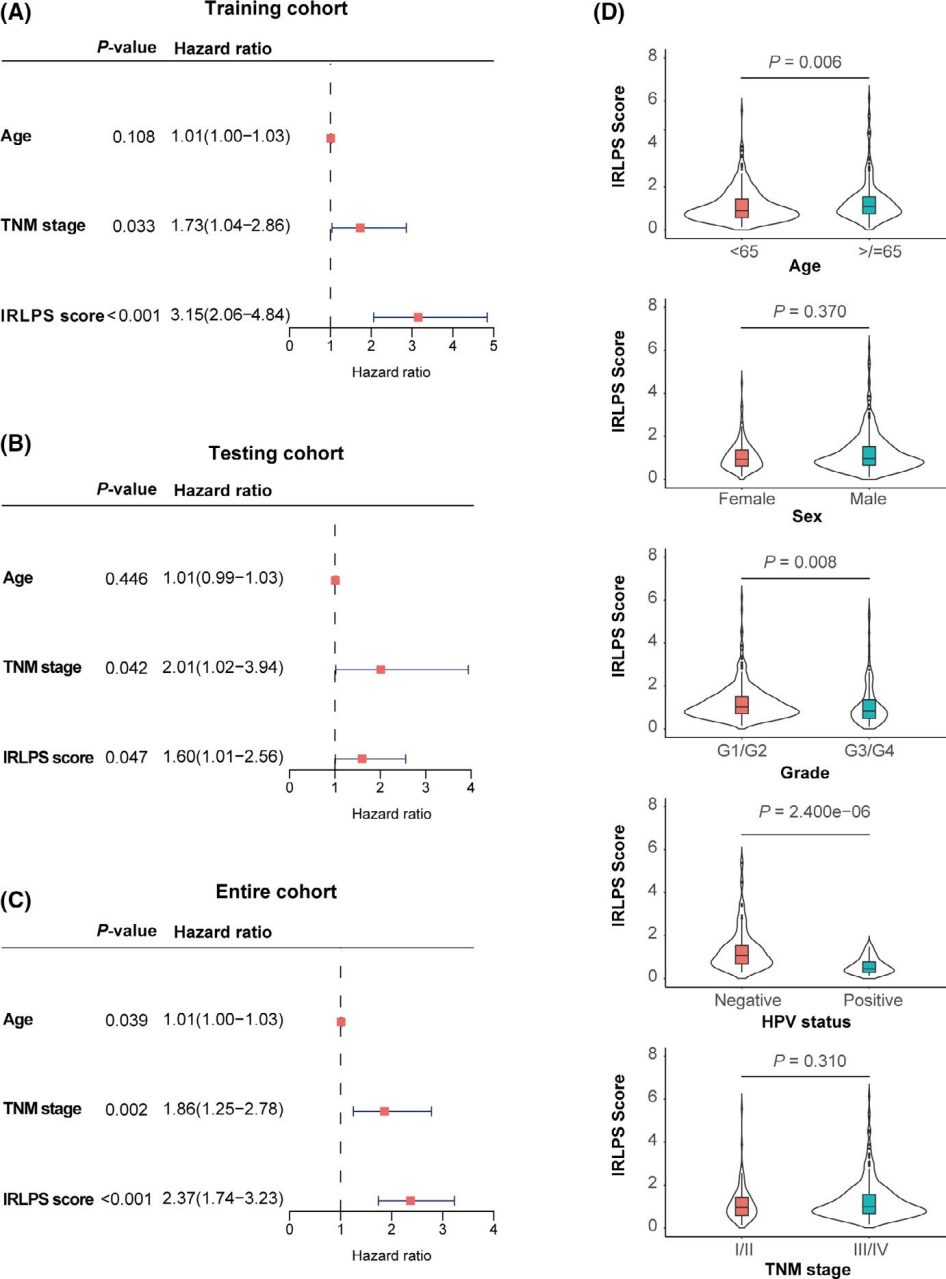
Independent prognostic factors for overall survival. Multivariate analysis of the (A) training cohort, (B) testing cohort and, (C) entire cohort. (D) Distribution of immune‐related lncRNA prognostic signature (IRLPS) scores in the different subgroups stratified by age, sex, grade, HPV status, and stage

In the univariate Cox analysis, only age, stage, and IRLPS score were identified as significant prognostic factors among the clinicopathological factors (Figure 5A–C). Stage and IRLPS scores were confirmed to be independently associated with OS time in the multivariate Cox analysis in the training cohort (Figure 6A). The results in the testing cohort (*p* = 0.042 and *p* = 0.047) (Figure 6B) and entire cohort (*p* = 0.002 and *p* < 0.001) (Figure 6C) were consistent with those in the training cohort.

In terms of the distribution of IRLPS score in the subgroups stratified by other clinicopathological factors, the IRLPS score was higher in patients in the older age group (*p* = 0.006), with low‐grade tumor (*p* = 0.008) and negative HPV status (*p* = 2.400 × 10^−6^), whereas there were no differences between the two IRLPS subgroups in terms of sex and TNM stage (Figure 6D).

### 3.5 Discriminability of IRLPS

**FIGURE 7:**
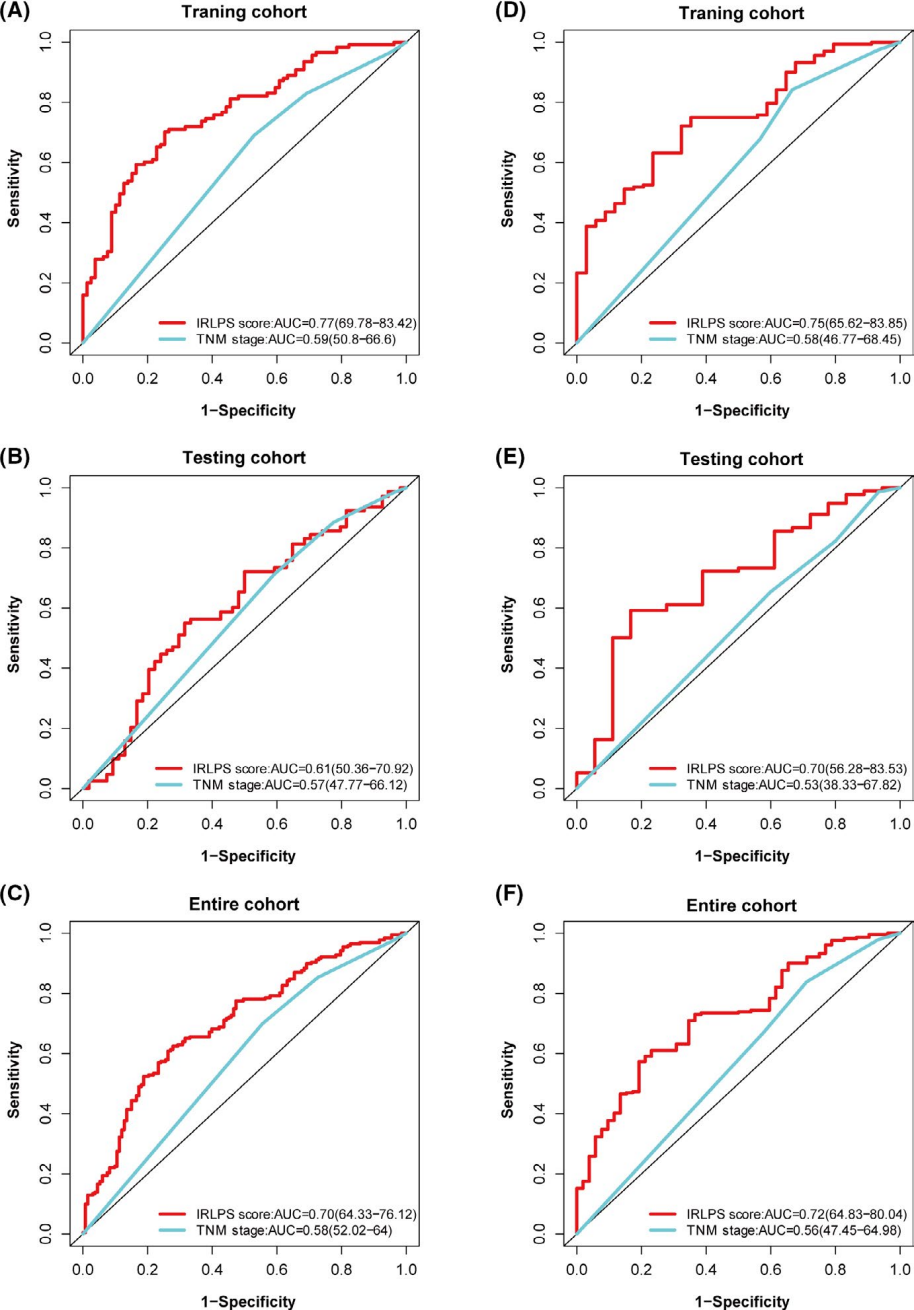
Discriminability of immune‐related lncRNA prognostic signature (IRLPS). ROC curves of IRLPS score and stage at the 3‐year follow‐up in the (A) training cohort, (B) testing cohort, and (C) entire cohort. The ROC curves of IRLPS score and stage at the 5‐year follow‐up in the (D) training cohort, (E) testing cohort, and (F) entire cohort

Based on the above results, IRLPS was identified as a prognostic biomarker of HNSCC; therefore, we further evaluated its discriminability compared with the TNM stage. In terms of IRLPS, the AUC values of the ROC curves in the training, testing, and entire cohorts were 0.77, 0.61, and 0.70, respectively, at the 3‐year follow‐up (Figure 7A–C), and 0.75, 0.70, and 0.72, respectively, at the 5‐year follow‐up (Figure 7D–F). In terms of TNM stage, the AUC values of the ROC curves in the training, testing, and entire cohorts were 0.59, 0.57, and 0.58, respectively, at the 3‐year follow‐up (Figure 7A–C), and 0.58, 0.53, and 0.56, respectively, at the 5‐year follow‐up (Figure 7D–F). Thus, the predictive accuracy of the signature lncRNAs was all higher than that of the TNM stage.

### 3.6 Survival analysis in different subgroups

**FIGURE 8:**
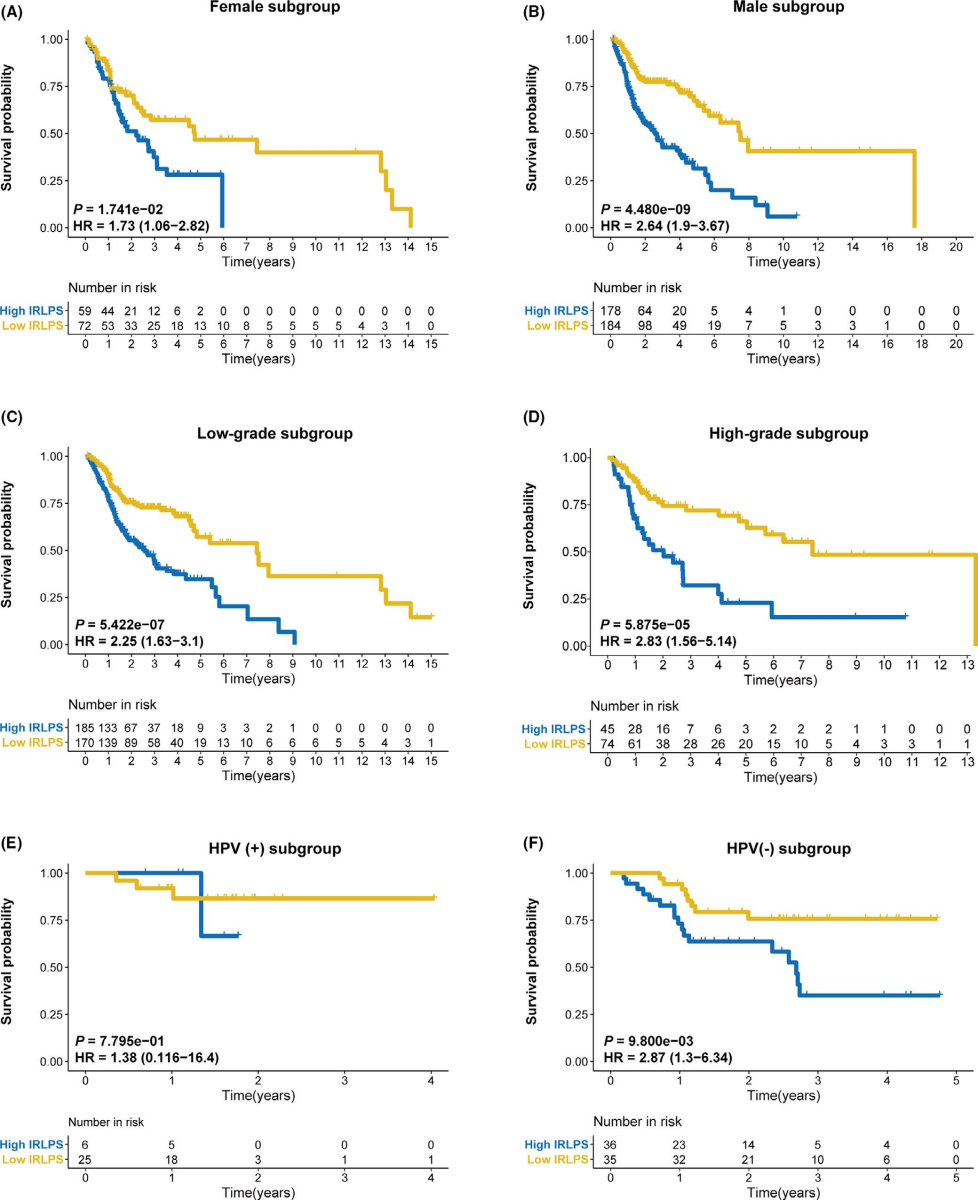
Subgroup analysis of immune‐related lncRNA prognostic signature (IRLPS) based on IRLPS. The Kaplan–Meier survival curves for overall survival in the high‐ and low‐IRLPS subgroups in the (A) female subgroup, (B) male subgroup, (C) low‐grade subgroup, (D) high‐grade subgroup, (E) HPV (+) subgroup, and (F) HPV(‐) subgroup

To further assess the prognostic value of IRLPS, we generated K–M curves and log‐rank tests in the subgroups. IRLPS was shown to distinguish the prognosis of female patients (*p* = 1.741 × 10^−2^) (Figure 8A), male patients (*p* = 4.480 × 10^−9^) (Figure 8B), grade_1–2_ patients (*p* = 5.422 × 10^−7^) (Figure 8C), grade_3–4_ patients (*p* = 5.875 × 10^−5^) (Figure 8D), and HPV(‐) patients (*p* = 9.800 × 10^−3^) (Figure 8F), but not HPV(+) patients (Figure 8E).

### 3.7 The difference of gene mutations in the two IRLPS subgroups

**FIGURE 9:**
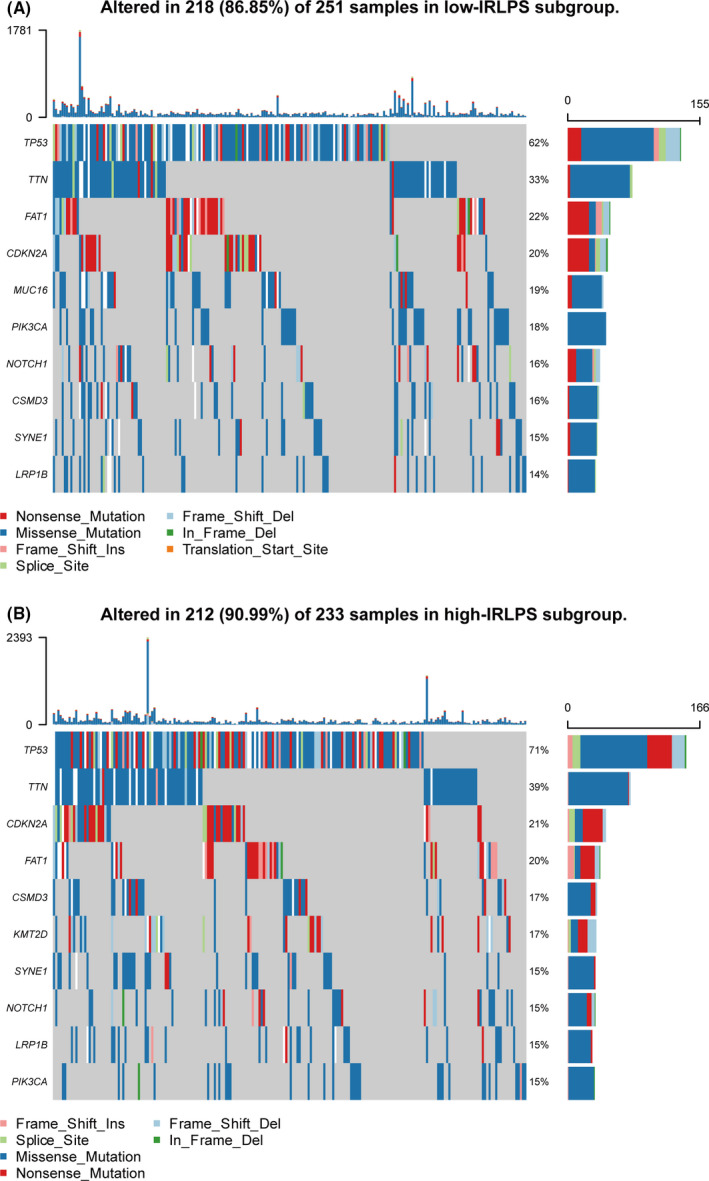
Mutation status of the high‐ and low‐immune‐related lncRNA prognostic signature (IRLPS) patients. (A) The top 10 genes with the highest mutation rate in the low‐immune‐related lncRNA prognostic signature (IRLPS) subgroup. (B) The top 10 genes with the highest mutation rate in the high‐IRLPS subgroup

Also, we study the mutation status of the HNSCC samples. As shown in Figure 9A,B, the most common type of mutation in HNSCC patients was missense mutation. Among the many mutant genes, the mutation rate of TP53 was significantly different between the two IRLPS subgroups. TP53 has been widely reported as a tumor suppressor gene and TP53 mutation has been widely confirmed to promote tumor progression and be related to poor prognosis.^20^, ^21^, ^22^ Consistent with our results, the high‐IRLPS patients with higher TP53 mutation (71%) had a worse outcome than the low‐IRLPS patients with lower TP53 mutation (62%).

### 3.8 The distribution of six immune cell types in the two IRLPS subgroups

**FIGURE 10:**
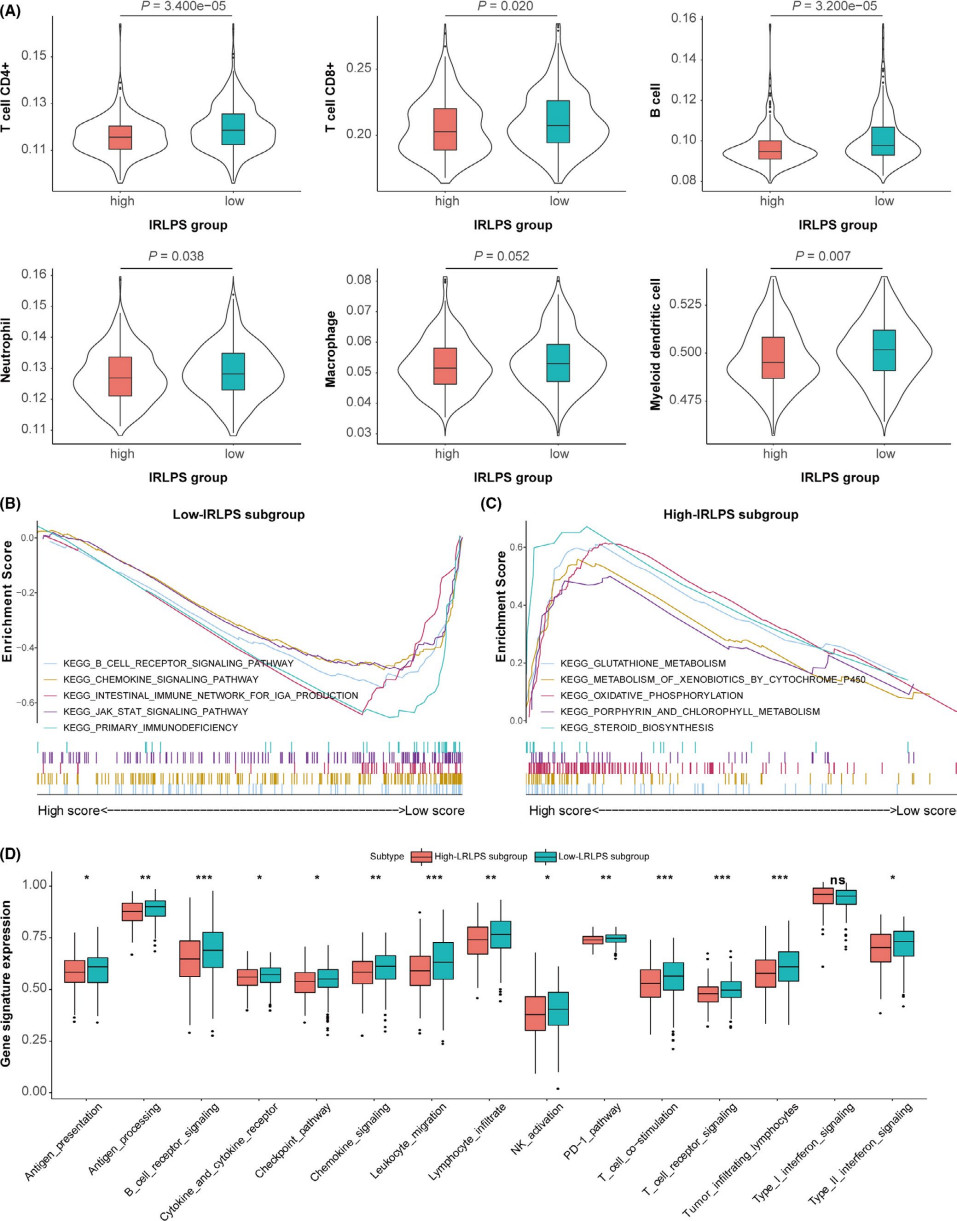
The tumor microenvironment characteristics of the high‐ and low‐immune‐related lncRNA prognostic signature (IRLPS) subgroups. (A) Infiltration by six immune cell types between the two IRLPS subgroups. (B, C) Functional enrichment in the two IRLPS subgroups according to gene set enrichment analysis. (D) The relative expression of some certain immune‐related gene signatures between the two IRLPS subgroups

To study the role of IRLPS in TME, we compared the infiltration by six immune cell types in the high‐ and low‐IRLPS subgroups. As shown in Figure 10A, CD4 T cells, CD8 T cells, B cells, neutrophils, and myeloid dendritic cells were significantly more common in the low‐IRLPS group. Macrophages were more common in the low‐IRLPS group than in the high‐IRLPS group, although the difference was not statistically significant.

### 3.9 Functional enrichment in two IRLPS subgroups

Functional enrichment scores were calculated by GSEA of the high‐ and low‐IRLPS subgroups based on IRLPS to identify the enriched GO biological processes and KEGG pathways (Detained in Table S2). In the low‐IRLPS group, we identified enhanced activity of some immune‐related pathways (*p* < 0.05) (Figure 10B), such as chemokine signaling pathway, B‐cell receptor signaling pathway, and JAK‐STAT signaling pathway. In contrast, metabolic pathways were more commonly enriched in the high‐IRLPS group (Figure 10C). To further define the immune function between two IRGPI subgroups, we performed ssGSEA on certain gene signatures and compared the score between two IRLPS subgroups, shown in Figure 10D. Compared with the high‐IRLPS subgroup, the low‐IRLPS subgroup was more enriched in antigen‐related gene sets, immune cell activation‐related pathways, checkpoint pathway, and specific cytokine pathways.^23^, ^24^

### 3.10 Construction and evaluation of the nomogram

**FIGURE 11:**
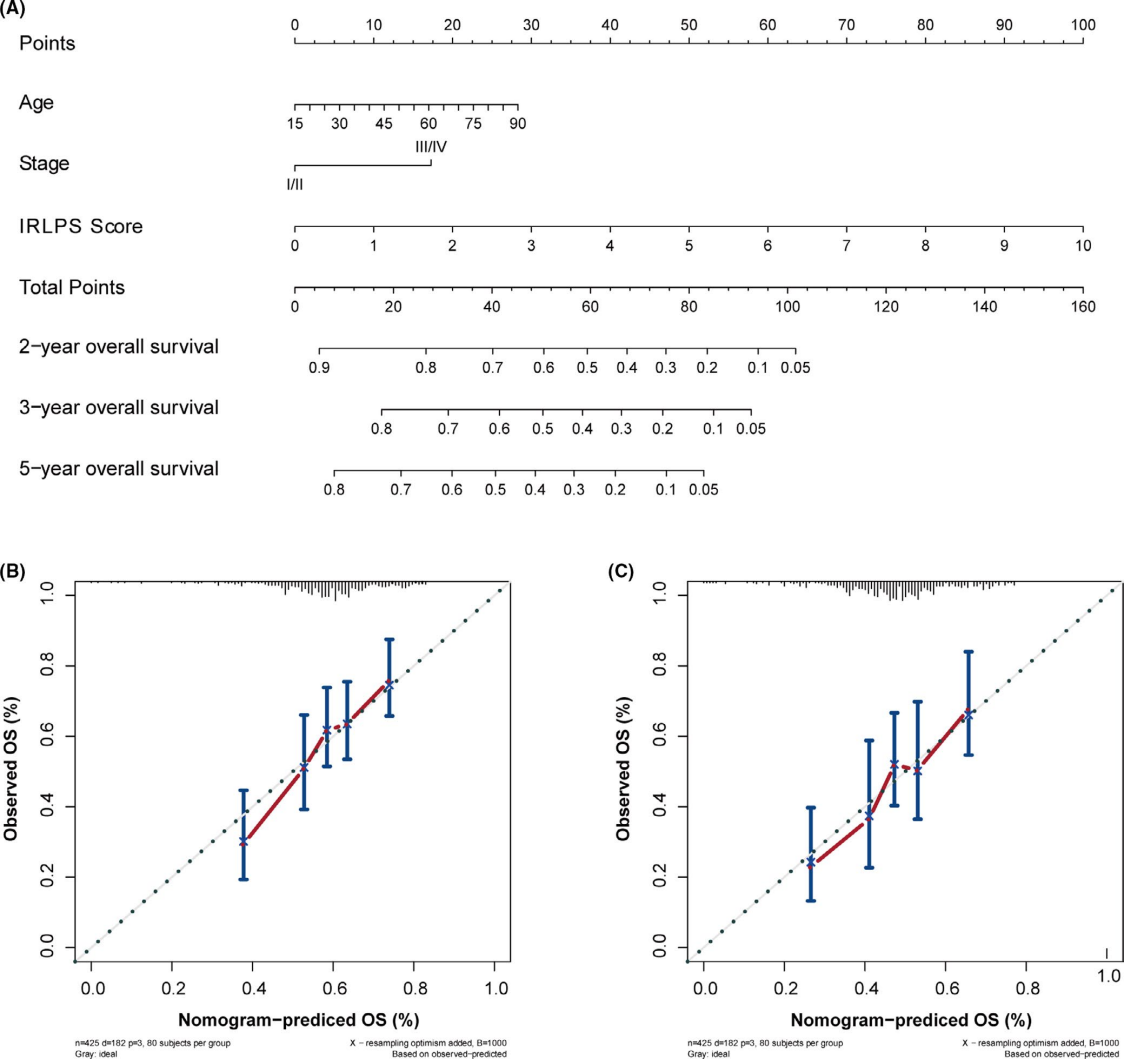
Nomogram construction. (A) Nomogram predictive of overall survival for head and neck squamous cell carcinoma (HNSCC). (B) Nomogram calibration plot at the 3‐year follow‐up. (C) Nomogram calibration plot at the 5‐year follow‐up

We finally constructed a nomogram to predict the 3‐ and 5‐year OS of HNSCC patients based on age, TNM stage, and IRLPS (Figure 11A). The calibration plots showed excellent agreement between predicted and observed outcomes at the 3‐ and 5‐year follow‐ups in all HNSCC patients (Figure 11B,C).

## 4 DISCUSSION

In our study, we identified an immune‐related lncRNA signature for HNSCC and evaluated its prognostic role in different cohorts and different subgroups. IRLPS had a better discriminability than the TNM stage. Stratified by the IRLPS score of IRLPS, low‐IRLPS patients had a better outcome than high‐IRLPS patients, had more infiltration of immune cells, and functional enrichment in immune‐related pathways.

Compared with ordinary genes, lncRNA has some unique characteristics and advantages. On one hand, the expression of lncRNA varies in different tissues, diseases, and the progression stage of the diseases. So the expression changes of lncRNA can better represent the specificity of a certain tissue or the special stage of the disease.^25^, ^26^, ^27^ On the other hand, lncRNAs are noncoding RNAs and directly involved in various biological processes, thus the levels and functions are more closely associated with the development characteristics of diseases including cancers.^25^, ^28^, ^29^, ^30^ At present, researches on immune‐related lncRNA in HNSCC are still lacking. This study can provide new ideas for the immune‐related role of lncRNA in HNSCC.

In our study, IRLPS was composed of seven lncRNAs, AL139158.2, AL031985.3, AC104794.2, AC099343.3, AL357519.1, SBDSP1, and AC108010.1. The pseudogene‐derived lncRNA SBDSP1 has been shown to suppress tumor growth and invasion and is related to poor outcomes in colorectal cancer.^31^, ^32^ We also identified six novel lncRNAs associated with HNSCC, which have not previously been discovered in recent explorations in humans.

After the development of IRLPS, we divided the HNSCC patients into high‐ and low‐IRLPS subgroups based on the IRLPS score of IRLPS. We then evaluated the prognostic value and discriminability of IRLPS in the two IRLPS subgroups. In the ROC analysis, the predictive accuracy of IRLPS was approximately 0.70 in the training, testing, or entire cohorts, while predictive accuracy of the TNM stage did not exceed 0.60, indicating that our model had a superior performance than TNM. Furthermore, the K–M survival curves of the training, testing, and entire cohorts and different subgroups supported the distinguishing ability of IRLPS. These results showed that IRLPS had a good degree of discrimination and is a promising prognostic biomarker.

In many other tumors, IRLPS has been confirmed as an excellent prognostic biomarker.^33^, ^34^ Furthermore, some studies showed that immune‐related lncRNA was not only a prognostic biomarker but can also be used to distinguish the characteristics of the TME.^14^, ^35^ The prognostic value of immune cell infiltration has been verified in a variety of solid tumors.^36^, ^37^, ^38^ Therefore, we further studied the TME in HNSCC. Comparison of the infiltration by six immune cell types (CD4 T cells, CD8 T cells, B cells, neutrophils, macrophages, and myeloid dendritic cells) showed that all six immune cell types were more densely distributed in the low‐IRLPS group than in the high‐IRLPS group. These findings suggested that low‐IRLPS patients might have more active immune responses, immune system processes, and related immune functions than high‐IRLPS patients. CD8 T cells are involved in cellular immune responses that are critical for antitumor immunity,^39^ and the presence of CD8 lymphocytes in the TME has been correlated with a better prognosis in various types of cancer.^40^, ^41^, ^42^ Due to the wide range of CD4 cell subsets with different functions,^43^ the role of CD4 T cells is unclear and its prognostic value is controversial.^44^ Interestingly, some studies have shown that B‐cell infiltration can predict a good prognosis in early stage HNSCC, while it is negatively correlated in the advanced stage, indicating that the function and composition of B cells are plastic during the disease process.^45^, ^46^ Macrophages are immune cells that produce proangiogenic and immunosuppressive factors. In most tumors, M2 macrophages are correlated with poor outcome, while M1 macrophages are associated with a favorable prognosis.^47^ Previous studies have found that neutrophils and myeloid dendritic cells contribute to the development of an immunosuppressive microenvironment and correlate with a poor prognosis^48^, ^49^ Although the degree of infiltration by some immune cells was correlated with a poor outcome, in general, the low‐IRLPS patients with more immune cell infiltration had a better prognosis, indicating that the TME is complicated and the distribution of certain immune cells alone does not allow for accurate prediction. In the GSEA analysis, we found numerous immune‐related signal pathways that were enriched in the low‐IRLPS group, which was consistent with the distribution of immune cells. To better understand why the outcome of high‐IRLPS and low‐IRLPS patients were different from the perspective of immunity, we performed ssGSEA between two IRLP subgroups. The low‐IRLPS subgroup was more enriched in antigen‐related gene sets, immune cell activation‐related pathways, checkpoint pathway, and specific cytokine pathways than the high‐IRLPS subgroup. Therefore, we inferred that low‐IRLPS patients could produce more antigens to activate the immune system, produce more active immune cells, and secrete more active cytokines, which might lead to a better prognosis. The results of ssGSEA were corresponding with the results of GSEA.

In this study, we developed a prognostic signature based on immune‐related lncRNAs to predict the prognosis of HNSCC patients, and they have been observed to be clinically relevant and effective in different data sets. To the best of our knowledge, this is the first report of an immune‐related lncRNA‐based signature in HNSCC. Nevertheless, there were several limitations to our study. For example, due to a lack of in vitro and in vivo studies, the TME and the molecular mechanisms of HNSCC could not be fully elucidated. Besides, the prognostic biomarker was not been tested and analyzed in clinical samples. Further studies are therefore warranted.

## 5 CONCLUSION

In this study, we developed and validated an immune‐related lncRNA signature that can be used to stratify HNSCC patients into high‐ and low‐IRLPS subgroups with distinct survival outcomes, for which dysregulation of TME might be responsible. These findings may provide insights into the development of novel immune‐related biomarkers.

## CONFLICT OF INTEREST

The authors have declared no conflict of interest.

## AUTHOR CONTRIBUTIONS

Conceptualization, Y.S., Y.P.M., L.L., and Y.C.; Data curation, T.Q.L. and S.S.X.; Investigation, S.S.X.; Methodology, T.Q.L. and Y.C.; Software, T.Q.L..; Writing – Original Draft Preparation, T.Q.L. and Y.C.; Writing – Review & Editing, Y.P.M., Y.C., and C.Y.C.; Visualization, T.Q.L.; Validation, Y.C. and Y.P.M.; Supervision, Y.S. and L.L.; Project Administration, Y.S., Y.P.M., and L.L.; Funding Acquisition, Y.S. and C.Y.C.

## Supporting information

Table S1

Table S2

## Data Availability

The data that support the findings of this study are available from the corresponding author upon reasonable request.
