# Supplementary material for: An immune‐related seven‐lncRNA signature for head and neck squamous cell carcinoma
Source: Cancer Med. 2021 Mar 3;10(7):2268–85. doi: 10.1002/cam4.3756 (PMC7982618; doi:10.1002/cam4.3756)
Supplement: Supplementary file 1 — Table S1 [file CAM4-10-2268-s001.docx]

| **Table S1. The details of the seven lncRNAs.** | | | | | |
| --- | --- | --- | --- | --- | --- |
| **Hg38_name** | **Ensemble_ID** | **Chr** | **Start** | **End** | **Strand** |
| AL139158.2 | ENSG00000224592 | Chr1 | 38047314 | 38119025 | + |
| AL031985.3 | ENSG00000260920 | Chr1 | 40464319 | 40466767 | + |
| AC104794.2 | ENSG00000260077 | Chr2 | 10039092 | 10040663 | - |
| AC099343.3 | ENSG00000271646 | Chr4 | 184474802 | 184477304 | + |
| AL357519.1 | ENSG00000230943 | Chr6 | 113623535 | 113650074 | - |
| SBDSP1 | ENSG00000225648 | Chr7 | 72829425 | 72836701 | + |
| AC108010.1 | ENSG00000242588 | Chr7 | 128574751 | 128622694 | + |
